# Clinical array-based karyotyping of breast cancer with equivocal HER2 status resolves gene copy number and reveals chromosome 17 complexity

**DOI:** 10.1186/1471-2407-10-396

**Published:** 2010-07-28

**Authors:** Shelly Gunn, I-Tien Yeh, Irina Lytvak, Budi Tirtorahardjo, Natasha Dzidic, Soheila Zadeh, Jaeweon Kim, Chris McCaskill, Lony Lim, Mercedes Gorre, Mansoor Mohammed

**Affiliations:** 1 Combimatrix Molecular Diagnostics, 310 Goddard, Irvine California, 92618, USA; 2 Department of Pathology, University of Texas Health Science Center at San Antonio, 7703 Floyd Curl, San Antonio, Texas 78229, USA; 3 START Center for Cancer Care, 4383 Medical Drive, San Antonio, Texas 78229, USA

## Abstract

**Background:**

*HER2 *gene copy status, and concomitant administration of trastuzumab (Herceptin), remains one of the best examples of targeted cancer therapy based on understanding the genomic etiology of disease. However, newly diagnosed breast cancer cases with equivocal HER2 results present a challenge for the oncologist who must make treatment decisions despite the patient's unresolved HER2 status. In some cases both immunohistochemistry (IHC) and fluorescence *in situ *hybridization (FISH) are reported as equivocal, whereas in other cases IHC results and FISH are discordant for positive versus negative results. The recent validation of array-based, molecular karyotyping for clinical oncology testing provides an alternative method for determination of HER2 gene copy number status in cases remaining unresolved by traditional methods.

**Methods:**

In the current study, DNA extracted from 20 formalin fixed paraffin embedded (FFPE) tissue samples from newly diagnosed cases of invasive ductal carcinoma referred to our laboratory with unresolved HER2 status, were analyzed using a clinically validated genomic array containing 127 probes covering the HER2 amplicon, the pericentromeric regions, and both chromosome 17 arms.

**Results:**

Array-based comparative genomic hybridization (array CGH) analysis of chromosome 17 resolved HER2 gene status in [20/20] (100%) of cases and revealed additional chromosome 17 copy number changes in [18/20] (90%) of cases. Array CGH analysis also revealed two false positives and one false negative by FISH due to "ratio skewing" caused by chromosomal gains and losses in the centromeric region. All cases with complex rearrangements of chromosome 17 showed genome-wide chromosomal instability.

**Conclusions:**

These results illustrate the analytical power of array-based genomic analysis as a clinical laboratory technique for resolution of HER2 status in breast cancer cases with equivocal results. The frequency of complex chromosome 17 abnormalities in these cases suggests that the two probe FISH interphase analysis is inadequate and results interpreted using the HER2/CEP17 ratio should be reported "with caution" when the presence of centromeric amplification or monosomy is suspected by FISH signal gains or losses. The presence of these pericentromeric copy number changes may result in artificial skewing of the HER2/CEP17 ratio towards false negative or false positive results in breast cancer with chromosome 17 complexity. Full genomic analysis should be considered in all cases with complex chromosome 17 aneusomy as these cases are likely to have genome-wide instability, amplifications, and a poor prognosis.

## Background

Overexpression of the HER2 protein in breast cancer is most often the result of *HER2 *gene amplification on the q arm of chromosome 17. Standard testing methods include analysis of HER2 protein expression on the cell membrane by IHC and/or evaluation of *HER2 *gene copy number by *in situ *hybridiztion (ISH), most commonly fluorescence (FISH), but also silver (SISH) or chromogenic (CISH), using DNA-based probes targeting the HER2 gene locus and chromosome 17 centromere (CEP) [1]. HER2 protein overexpression and gene amplification are prognostic markers for aggressive tumors and predictive of response to the drugs trastuzumab (Herceptin^®^) and lapatinib (Tykerb^®^). Accurate and definitive reporting of HER2 status is thus essential for appropriate treatment planning in newly diagnosed cases. Yet despite the clinical need for accurate determination of HER2 status, it is estimated that approximately 20% of current HER2 testing results may be inaccurate. This inaccuracy has been most often attributed to multiple preanalytic, analytic, and postanalytic variables inherent to the mechanics of performing the test in a clinical laboratory [2].

In addition to testing inaccuracies, clinicians are also faced with treatment dilemmas resulting from cases that are reported as "equivocal" after testing by IHC and FISH have been completed. These cases are either 2+ by IHC and/or have a HER2/CEP17 ratio between 1.8 and 2.2. However, there are also instances where the results of FISH and IHC are discordant such that one test is reported as positive (amplified) and the other as negative (unamplified). Although the numbers of these equivocal and discordant cases vary widely between laboratories, it is estimated in some studies to be as high as 20% of cases [3].

The prevalence of inaccurate, discordant, and equivocal HER2 results has lead to a reexamination of the adequacy of existing methods to accurately detect copy number changes involving the *HER2 *gene, particularly in the setting of complex chromosome 17 rearrangements. Recent genome wide array CGH studies have revealed that complete polysomy 17, which had previously been reported as prevalent in breast cancer, is actually a rare event [4,5]. These and similar extended FISH studies of chromosome 17 in breast cancer have additionally shown that amplifications of the pericentromeric region are common occurrences in both HER2 positive and HER2 negative cases [6,7]. The complexity of these chromosome 17 pericentromeric rearrangements detected by both array CGH and FISH analysis has brought into question the accuracy of reported HER2/cep 17 ratios in cases where complex segmental aneusomy of chromosome 17 is present. This observation has lead to the hypothesis that unsuspected chromosome 17 copy number changes may be contributing to the high percentage of inaccurate and equivocal results for HER2 status in breast cancer.

The recent introduction of array-based molecular karyotyping into some clinical laboratories provides an alternative method for clinical genomic evaluation of oncology samples [8,9]. Array-based chromosomal analysis combines the precision of locus-specific FISH with a complete, whole-genome view of the chromosome complement of a cell, giving clinicians both an accurate assessment of copy number changes involving specific genes, as well as an evaluation of the relevant chromosomes in the context of the entire genome. There is growing evidence that copy number evaluation of *both *the *HER2 *gene and chromosome 17 are of prognostic significance in breast cancer, and that aneusomy 17 with or without *HER2 *gene amplification is associated with poor prognostic factors [10-12].

The aim of this study was to resolve the *HER2 *gene and chromosome 17 status in cases of invasive ductal carcinoma, where gene copy number and chromosome status were equivocal and/or discordant (based on previously performed IHC and FISH analysis), by using a clinical array CGH assay for copy number evaluation of multiple loci along chromosome 17.

## Methods

### DNA Extraction from FFPE Tissues

DNA was extracted from formalin fixed, paraffin embedded (FFPE) tumor tissues in 20 de-identified, cases of invasive ductal carcinoma with previously evaluated and unresolved HER2 and/or chromosome 17 status due to suspected pericentromeric aneusomy (n = 2), discordance (n = 2), and equivocal results (n = 16). Tumors were visualized and marked on H&E stained sections cut from the FFPE block, with targeted areas captured by slide scraping or 4 mm punch as described previously [13]. Following proteinase K digestion, a minimum of 2 μg DNA was isolated using the Promega Maxwell 16, with verification of high molecular weight DNA by agarose gel electrophoresis.

### Array-based genomic analysis

**Figure 1:**
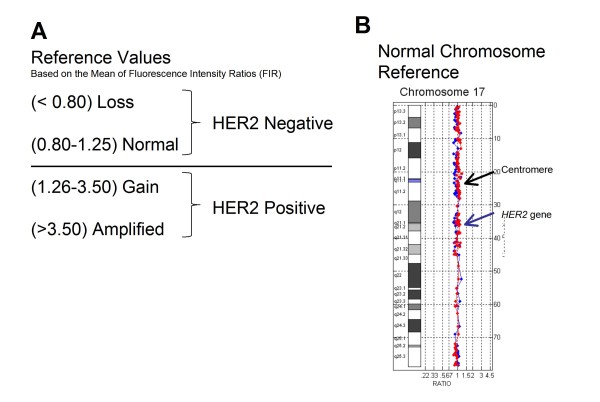
**Reference Values**. A: Objective reference values for determination of *HER2 *gene copy number, B: Subjective determination by visualization of chromosome 17 ratio plot.

Array CGH analysis of the tumor genome was performed at Combimatrix Molecular Diagnostics (CMDX) (Irvine, CA, USA) using the Her17Array™ test, a 3039 probe whole genome bacterial artificial chromosome (BAC) microarray with 127 probes targeting the *HER2/TOP2A *amplicon, the pericentromeric regions and both chromosome 17 arms. Tumor genomic DNA (test DNA) and male reference DNA (as an internal control) were differentially labeled with Alexa Fluor 555 and Alexa Fluor 647 fluorescent dyes (Life Technologies, Carlsbad, CA, USA) in dye swap reactions and hybridized to the BAC arrays including four overlapping probes for the *HER2 *gene, (RP11-62N23, G248P82514H9, RP11-1065L22, RP11-94L15) and 123 additional probes for coverage of chromosome 17, the pericentromeric region (base pairs 21.72-23.39 Mb) and the HER2/TOP2A amplicon (base pairs 34.73-36.54 Mb and genes *PPARBP, PPP1R1B, STARD3, TCAP, PNMT, PERLD1, GRB7, GSDML, PSMD3, CASC3, RARA, TOP2A, SMARCE1*) [14]. Hybridized microarray slides were scanned and quantified with GenePix 4000B scanner and GenePix Pro (Molecular Devices, Sunnyvale, CA, USA). The normalized Alexa Fluor 555/647 intensity ratios were computed for each of the two reactions and plotted together for each chromosome using internally developed software (Combimatrix Molecular Diagnostics, Irvine, CA, USA). A ratio plot was assigned such that gains in DNA copy number at a particular locus are observed as the simultaneous deviation of the ratio plots from a modal value of 1.0, with the blue ratio plot showing a positive deviation (to the right) while the red ratio plot shows a negative deviation at the same locus (to the left). HER2 positive versus negative status was subjectively determined by ratio plot visualization and objectively determined by calculating the mean of the fluorescence intensity ratio (FIR) values for the *HER2 *gene probes. A value > 1.25 was scored as positive (amplified) and ≤ 1.25 negative (unamplified) (Figures 1a and 1b, Table 1).

**Table 1:** Breast cancer cases with equivocal HER2 status.

Case	Reason for Referral	HER2 status by array CGH	FIR Value
EQ-1	Equivocal by FISH; Polysomy?	Negative	1.18
EQ-2	Equivocal by IHC and FISH	Positive	1.47
EQ-3	Equivocal by IHC and FISH	Positive	2.36
EQ-4	Equivocal; HER2 polyploidy?	Negative	1.09
EQ-5	HER2+; HER2/centromere co-amplification?	Positive	3.06
EQ-6	Equivocal by FISH pericentromeric monosomy?	Negative	1.23
EQ-7	Equivocal by FISH; aneusomy?	Negative	1.19
EQ-8	Equivocal by FISH; aneusomy?	Negative	0.81
EQ-9	Equivocal by FISH; aneusomy?	Negative	1.00
EQ-10	Equivocal by IHC and FISH	Negative	0.92
EQ-11	Equivocal by IHC and FISH	Negative	1.02
EQ-12	HER2+; centromeric amplification?	Positive	4.23
EQ-13	HER2+ by IHC; polysomy?	Positive (false - by FISH)	1.35
EQ-14	Equivocal	Negative	0.81
EQ-15	Equivocal by IHC and FISH	Negative	1.00
EQ-16	Discordant: IHC 1+, FISH 2.86	Negative (false + by FISH)	0.97
EQ-17	2.7 "interpret with caution due to monosomy 17"	Negative (false + by FISH)	1.15
EQ-18	Resolve equivocal IHC, FISH, and OncotypeDX^®^	Positive	1.67
EQ-19	Equivocal by IHC and FISH	Negative	0.97
EQ-20	Resolve equivocal IHC, FISH and OncotypeDx^®^	Positive	3.70

Reason for referral and array CGH findings are summarized in twenty cases of invasive ductal carcinoma. HER2 and chromosome 17 status were determined by array CGH as determined by fluorescence intensity ratio (FIR) value. Two cases (EQ 18 and EQ 20) were also equivocal by OncotypeDX^® ^(Genomic Health, Redwood City, CA).

## Results

### Cases resolved by array CGH analysis

*HER2 *gene and chromosome 17 status were resolved in 20/20 (100%) of cases. Seven cases were HER2 positive by array CGH and 13 cases were HER2 negative. There were two cases with normal chromosome 17, one case of monosomy 17, and one case with *HER2 a*mplified polysomy 17. All other cases were found to have chromosome 17 rearrangements involving either or both chromosome arms and the pericentromeric regions. All cases could be resolved by subjective visualization of the chromosome 17 ratio plots, and objectively using the forward reaction mean of the FIR values for the HER2 probes (Table 1).

### HER2 positive cases and HER2 positive polysomy 17

**Figure 2:**
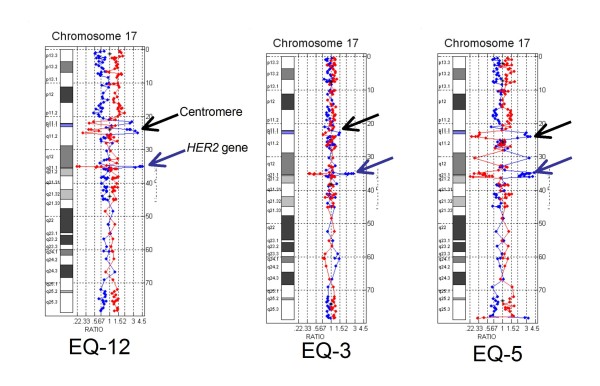
**HER2 positive cases with ratio-skewing by FISH**. HER2 positive cases showing co-amplification of the centromere and HER2 gene.

**Figure 3:**
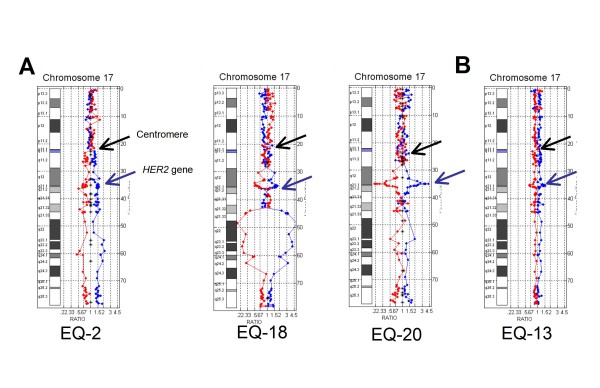
**HER2 positive cases with aneusomy and polysomy**. A: HER2 positive cases with complex chromosome 17 rearrangements B: Case of HER2 positive polysomy 17 which was positive by IHC and negative by FISH.

Of the cases that were HER2 positive by array CGH, three showed concurrent centromeric and *HER2 *gene amplification (Figure 2), and three had complex rearrangements involving the centromere (Figure 3A). In one case low level *HER2 *amplification was accompanied by chromosome 17 polysomy (Figure 3B). True unamplified polysomy 17 was distinguished from HER2 positive polysomy through subjective visualization of a greater simultaneous deviation of probes at the HER2 locus, and objectively by FIR values with numerical gains of the fluorescence intensity values for the *HER2 *gene that were greater than the fluorescent intensity values for the proximal and distal 17q probes.

### HER2 negative cases

**Figure 4:**
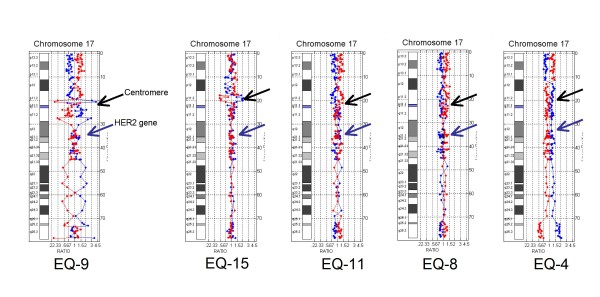
**HER2 negative cases with aneusomy**. HER2 negative cases showing complex pericentromeric rearrangements.

**Figure 5:**
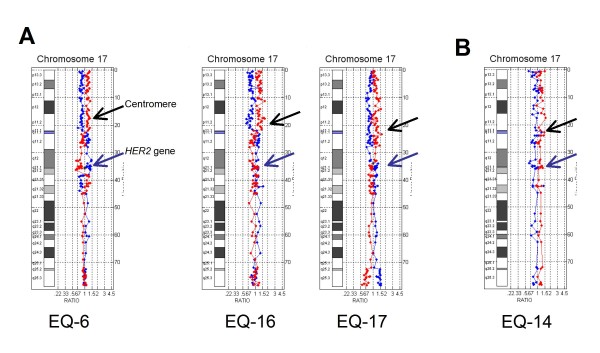
**HER2 negative cases with aneusomy and monosomy**. A: HER2 negative cases with centromeric loss B: Case showing complete monosomy 17.

Of the 13 HER2 negative cases by array CGH, five harbored complex chromosome 17 rearrangements (Figure 4) and three showed centromeric losses (Figure 5A). These cases with pericentromeric losses had been "reported with caution" as HER2 positive (n = 1) and equivocal (n = 2) by FISH due to skewing of the HER2/CEP17 ratio by centromeric monosomy. The remaining HER2 negative cases had non-amplified partial polysomy 17 (n = 1), normal chromosome 17 (n = 2), and one case of complete monosomy 17 (Figure 5B).

### Chromosome 17 findings correlated to genomic instability

Genome wide array-based chromosome analysis of the 18 cases positive for chromosome 17 abnormalities revealed high genomic instability (> 10 significant chromosomal aberrations) in 12 (67%) of cases and moderate to low genomic instability (5-10, and < 5 aberrations) in the other six cases. The two cases with normal chromosome 17 findings had low and moderate genomic instability.

## Discussion

In this study we used a clinically validated array CGH assay to resolve *HER2 *gene copy number and chromosome 17 status in 20 cases of breast carcinoma with equivocal or discordant results by IHC and FISH. HER2 status was determined subjectively and objectively as positive or negative in all cases and array-based testing additionally revealed a high incidence of chromosome 17 copy number changes involving both chromosome arms and the centromeric region. In most cases it could be determined that equivocal, false positive, or false negative FISH results were due to the complexity of the chromosome 17 abnormalities which had caused skewing of the HER2/CEP17 ratio. Artificial skewing of the Her2/CEP17 ratio occurs when the expected diploid number of the chromosome 17 centromere probe (D17Z1) is increased by centrome amplification or decreased by centromere loss. Chromosome 17 centromere amplification has been well described [15,16] and recently identified as a biomarker for adjuvant anthracycline benefit in early breast cancer [17]. Centromere amplification was detected in three cases in this study: case EQ-3 which was equivocal by FISH and cases EQ-5 and EQ-12 which were positive by FISH but had unresolved chromosome 17 status due to significant amplification of the D17Z1 probe.

Centromeric loss was identified by array CGH in four cases; three of these cases (EQ-6, EQ-16, EQ-17) showed complete p arm loss with no loss of 17q, and one case (EQ-14) had complete monosomy 17. This is to our knowledge, the first report of loss of the chromosome 17 centromere in breast cancer. However, many pathologists already recognize that CEP17 probe loss will artificially skew the HER2 ratio towards positive and an increasing number of cases, HER2 positive by FISH, are being reported "with caution" when monosomy 17 is present. For example, Case EQ-17 was reported as HER2 positive "with caution" due to the presence of monosomy 17. Case EQ-16 was called positive by FISH but discordant with IHC results that were negative (1+) for HER2 protein expression. Array CGH showed no amplification of the *HER2 *gene in either of these cases and confirmed that the FISH results were false positives due to ratio skewing caused by centromeric losses.

**Figure 6:**
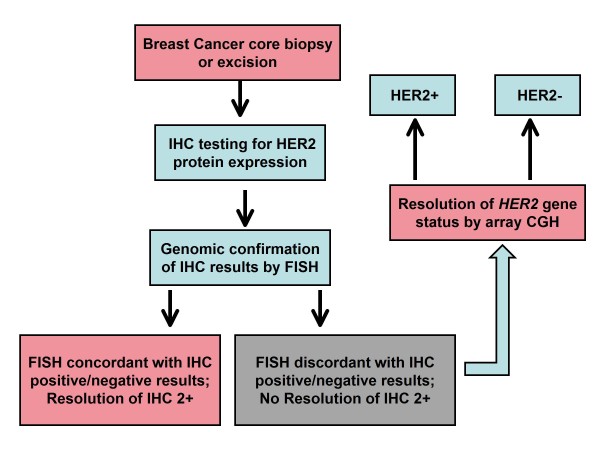
**Proposed algorithm for establishment of HER2 status in breast cancer samples by protein expression and genomic analysis**. IHC studies for HER2 protein expression are performed as part of the diagnostic workup and confirmed or resolved by FISH. If FISH and IHC results are concordant, or equivocal IHC results are resolved by FISH, no further testing is done. Equivocal cases by FISH and cases with discordant IHC/FISH results are resolved by array CGH.

College of American Pathologists/American Society of Clinical Oncology (CAP/ASCO) guidelines dictate use of the HER2/CEP17 ratio for reporting HER2 gene status by FISH as a correction for cases where polysomy 17 is present. However, recent array-based and extended FISH studies of chromosome 17 have shown that true polysomy 17 is an exceedingly rare event in breast cancer, and this was true not only in this study but also in previous studies by our laboratory where no cases of true polysomy 17 were detected [4]. However in this study, *HER2 *amplified polysomy 17 was identified by array CGH in a discordant case (EQ-13) which was HER2 positive by both array CGH and IHC (3+) and false negative by FISH due to skewing the HER2/CEP17 ratio (Figure 3B). It has been proposed, and is becoming increasingly clear in practice, that correction of *HER2 *gene FISH analysis with CEP17 probes may provide misleading HER2 gene status results in cases with centromeric gains and losses [15]. An alternative testing algorithm is therefore used by our laboratory which encompasses the CAP/ASCO guidelines but reflexes cases with discordant and/or equivocal results by IHC and FISH to array-based molecular karyotyping (Figure 6). Application of this algorithm is available to all pathologists, including those practicing in most hospital or community pathology labs, through technical only services offered by referral center laboratories. "Technical only" programs allow pathologists to interpret and bill for the professional component of array CGH testing without having to commit to the high expenses associated with bringing the test in-house.

## Conclusion

The ability of array-based molecular karyotyping assays to analyze the entire length of chromosome 17, in parallel with the HER2 gene locus, gives this newly introduced clinical laboratory technique a distinct advantage over FISH-based testing for determination of HER2 status. Detection of genomic changes involving chromosome 17 is clinically relevant not only for accurate HER2 status determination but also an important independent prognostic marker for aggressive disease. Analysis of chromosome 17 increases the accuracy of HER2 testing and simultaneously provides additional prognostic information about the tumor. In this study, taken together, complex aberrations of chromosome 17 was also found to be a predictor of high-risk cases likely to have additional genomic instability and gene amplifications, well recognized biomarkers for aggressive tumor behavior and overall reduced survival in breast cancer [18,19]. However, assessment of these markers has not heretofore been included as part of the diagnostic and prognostic patient workup because the clinical tools were not available to perform high resolution genomic analysis of FFPE tumor tissue in the clinical laboratory. With the introduction of array-based molecular karyotyping, *HER2 *gene and chromosome 17 status can now be accurately determined at the time of diagnosis and full genome analysis can be considered for high-risk patients who have tumors harboring complex chromosome 17 rearrangements.

## Competing interests

SG, BT, ND, SZ, JK, CM, LL, MG and MM are all employees of CMDX. IY and IL have no competing interests to declare.

## Authors' contributions

MM conceived and designed the array platform, MG and CM designed the array content and clinical assay, ND and SZ made the arrays, BT supervised the clinical team who ran the arrays, JK designed the software and analyzed the data, IY and IL analyzed clinical data and critically reviewed the manuscript, and SG helped draft the manuscript. All authors read and approved the final manuscript.

## Pre-publication history

The pre-publication history for this paper can be accessed here:

http://www.biomedcentral.com/1471-2407/10/396/prepub
